# Sleeping more than 8 h: a silent factor contributing to decreased muscle mass in Chinese community-dwelling older adults

**DOI:** 10.1186/s12889-024-18520-y

**Published:** 2024-05-06

**Authors:** Qiongyu Huang, Hongsheng Lin, Han Xiao, Liuwei Zhang, Dafang Chen, Xiaotong Dai

**Affiliations:** 1 Key Laboratory of Sports and Physical Health, Ministry of Education, Beijing Sport University, No.48, Xinxi Road, 100084 Beijing, China; 2 Department of Epidemiology and Biostatistics, School of Public Health, Peking University, No.38 Xueyuan Road, 100191 Beijing, China; 3 Key Laboratory of Epidemiology of Major Diseases (Peking University), Ministry of Education, Beijing, China

**Keywords:** Sleep duration, Low muscle mass, Generalized estimating equations, Longitudinal research

## Abstract

**Background:**

Muscle mass loss is an age-related process that can be exacerbated by lifestyle, environmental and other factors, but can be mitigated by good sleep. The objective of this study was to investigate the correlation between varying time lags of sleep duration and the decline in muscle mass among individuals aged 60 years or older by using real-world health monitoring data obtained from wearable devices and smart home health monitoring devices.

**Methods:**

This study included 86,037 observations from 2,869 participants in the Mobile Support System database. Missing data were supplemented by multiple imputation. The investigation utilized generalized estimating equations and restricted cubic spline curve to examine the relationship between sleep duration and low muscle mass. Various lag structures, including 0, 1, 2, 0–1, 0–2, and 1–2 months, were fitted, and the interaction effect of observation time with sleep duration was estimated for each lag structure. Additionally, subgroup analyses were conducted. The models were adjusted for various covariates, including gender, age, body mass index, footsteps, smoking status, drinking status, marital status, number of chronic diseases, number of medications, diabetes mellitus, hyperlipidemia, coronary artery disease, respiratory disease, and musculoskeletal disease and an interaction term between time and sleep duration.

**Results:**

The results of the generalized estimating equation showed a significant correlation (*p* < 0.001) between sleep duration of 8 h or more and low muscle mass in older adults, using 6–7 h of sleep as a reference. This effect was seen over time and prolonged sleep accumulated over multiple months had a greater effect on muscle mass loss than a single month. The effect of long sleep duration on muscle mass loss was significantly greater in females than in males and greater in the over-75 than in the under-75 age group. Restricted cubic spline plots showed a non-linear relationship between sleep duration and low muscle mass (*p* < 0.001).

**Conclusions:**

This study found an association between sustained nighttime sleep of more than eight hours and decreased muscle mass in older adults, especially older women.

**Supplementary Information:**

The online version contains supplementary material available at 10.1186/s12889-024-18520-y.

## Introduction

Over the course of a person’s life, age-related loss of muscle mass begins in middle age and decreases at a rate of 1% per year [1]. A longitudinal study [2] found that the rate of muscle mass loss was 0.8-0.98% for men and 0.64-0.7% for women over the age of 75. Loss of muscle mass with age leads to reduced muscle strength and limited physical function and is a significant risk factor for disability and death [3, 4]. In addition to the natural physiological process of aging [1, 5, 6], other factors [6] such as lifestyle choices [6], environment conditions, dietary habits [7, 8], and chronic diseases [9] accelerate the loss of muscle mass.

Both a prospective cohort [10] and a longitudinal study [11] had shown a U-shaped relationship between sleep duration and mortality in people over the age of sixty. Nocturnal sleep patterns in older adults change with age [12], and being sedentary or bedridden [13]can lead to disrupted circadian rhythms [12, 14], which, if maintained over time, can have a negative impact on the health of older adults [12, 15].

Sleep is recognized as an important factor in maintaining muscle mass [16].Several cross-sectional studies [17, 18] had demonstrated an association between too much or too little sleep and decreased muscle mass in older adults. The validity of research hypotheses requires evidence to further demonstrate causality, but there are fewer longitudinal studies [19–21]in this area. The results of related studies suggested that sleep duration affects muscle mass decline in older adults, and gender differences in the effect of sleep duration on muscle mass decline have not been consistent across these studies.

Therefore, based on real-world collected data, this study explored the longitudinal associations between sleep duration and low muscle mass in older adults, as well as differences in effects across age and gender.

## Methods

### Study design and participants

The dataset used in this study was extracted from a database based on a mobile support system [22]. It recorded basic user information and health-related observations of older adults aged 60 years or older recruited from communities in five Chinese cities (Taiyuan, Jinan, Nanchang, Guangzhou, and Hefei) between January 1, 2018, and September 31, 2022. The exclusion criteria for the data were as follows: (1) age less than 60 years; (2) the upload to the database was not in the above time period; (3) exclusion of participants with less than 1 sleep duration or low muscle mass data; (4) excluded indicators with a relatively large degree of deficiency. We preprocessed the collected data by logistic checking and outlier checking and computed the average observations per user per month. A total of 86,037 observations from 2,869 participants were included in the statistical analysis. All study procedures were approved by the Ethical Review Committee of Peking University. Informed consent was obtained from all individual participants included in the study.

### Data collection

#### Measurements and definitions

Participants were required to wear an exercise bracelet daily to monitor daily steps and sleep. Height was measured with a height and weight meter (HDM-300; Zhejiang Huaju Company, China) [22], which required light clothing, no shoes, and standing. Body fat weight was measured with a multi-frequency bioelectrical impedance analyzer (InBody 720; Biospace, Seoul, Korea) with 8-point tactile electrodes [23]. Before the test, the subject should fast or empty stomach for more than two hours, avoid drinking a lot of water and strenuous exercise, remove metal objects and wear single clothes. During the measurement, the subject stood barefoot on the electrode, entered the subject’s information according to the prompts, then held the handle with both hands and lifted it upward, with both arms naturally hanging down at 30° to the body, and stood still for the measurement. The whole measurement time is about 1–2 min.

Sleep duration was categorized into 4 groups: <6 h for insufficient sleep [24], 6–7 h for reference, 7–8 h, and ≥ 8 h for excessive sleep [17]. Fat-free mass index (FFMI) was calculated as fat-free mass (kg) divided by the square of height. Low muscle mass was defined [25] as FFMI < 16.31 kg/m² for men and FFMI < 13.96 kg/m² for women.

The data from each measurement taken by the participants were automatically uploaded via Bluetooth to an application on their cell phones and stored to the cloud-system [22].

#### Covariates

Baseline demographic information on participants was collected through a questionnaire (Supplementary questionnaire). Smoking status was categorized into never smokers and smokers (including current and former smokers); drinking status was categorized into never drinkers and drinkers (including current and former drinkers); marital status was categorized into single (including divorced, widowed, and separated) and married; and the number of medications [19] was categorized into < 5 and ≥ 5. We calculated the number of chronic conditions and categorized them as 0 (reference value), 1, 2, and ≥ 2. Daily steps [26] was considered as an indicator to assess physical activity and categorized into 3 classes, < 4000 steps, 4000–8000 steps, > 8000 steps (reference value). BMI (kg/m²) was calculated by weight/height² and referred to the GLIM [27] criteria for the use of BMI as an indicator for the assessment of nutritional status: <18.5 kg/m² was defined as malnutrition.

### Statistical analysis

Differences in demographic characteristics between participants with and without low muscle mass at baseline values were compared. Continuous variables were expressed as mean and standard deviation (SD) and analyzed by Student’s t-test. For categorical variables, correlation analyses were performed using χ² when expressed as counts (%).

We imputed missing values using a fully conditional specification with multiple imputation, using logistic regression for categorical variables and regression for continuous variables. We included daily steps, BMI, smoking status, marital status, number of chronic diseases, number of medications taken, diabetes, coronary heart disease, respiratory disease, and musculoskeletal disease as auxiliary variables in the imputed model. Drinking status, sex, age, observation time, hyperlipidemia, and an interaction term between sleep duration and observation time (but not included in the imputation model) were included in the statistical analysis model based on the imputed model. The distributions of the variables were compared between the different populated and original data sets. Regression coefficients were summarized using Rubin’s Rule. Sensitivity analyses were performed by comparing the Odds ratio and 95% confidence intervals as well as the *p*-values between the original data set and the imputed composite results.

Generalized estimating equations were used to assess the association between sleep duration and low muscle mass in different lag structures. We fitted different lag structures from the current month (lag0) to three lag months (lag3) as well as two months (lag0-1, 1–2) and three months (lag0-2). We examined the interaction of sleep duration with observation time and modeled three models in each lag structure. In Model A, sleep duration was not adjusted for other covariates. In Model B, age, gender, and body mass index were added to adjust for sleep duration. Model C adjusted Model B by adding smoking status, drinking status, marital status, number of daily steps, number of chronic diseases, number of medications, diabetes, hyperlipidemia, coronary heart disease, respiratory disease, and musculoskeletal disease. Stratified analyses were conducted among older adults of different genders (female and male) and age groups (60–65 years and ≥ 75 years). Restricted cubic spline curve was also used to examine nonlinear trends between sleep duration and low muscle mass in different lag structures.

All analyses were performed by SAS (version 9.4, SAS Institute Inc, Cary, CN, USA) and R, version 4.3.2 (R Project for Statistical Computing). *P* < 0.05 was considered statistically significant.

## Results

### Participants

Differences between low and non-low muscle mass participants at baseline was demonstrated in Table 1. The statistical analysis culminated in the use of 86,037 records from 2869 participants aged 60 years or older. Of these, 70.79% were female, with a mean age of (72.39 ± 6.37) years and a mean duration of observation of (29.99 ± 17.24) months. Compared with non-low-muscle-mass participants, low-muscle-mass participants were more likely to have the following characteristics: lower BMI, an average of fewer than 4,000 steps per day, and 2 or more chronic diseases.

**Table 1:** Characteristics of participants between low muscle mass and non-low muscle mass at baseline

Characteristics	Overall (*n* = 2869)	Low muscle mass(*n* = 55)	Non-low muscle mass(*n* = 1304)	*p* value
Sex, *N* (%)				< 0.001^#^
Female	2031(70.79)	19(34.55)	952(73.01)	
Male	838(29.21)	36(65.45)	352(26.99)	
Age, year, mean (SD)	72.39(6.37)	72.509(5.83)	71.56(6.37)	0.278*
Observation time, month, mean (SD)	29.99(17.24)	18.436(10.57)	16.643(10.26)	0.205*
Sleep duration, *N* (%)				< 0.001^#^
< 6 h	317(11.05)	2(3.64)	154(11.81)	
6–7 h	689(24.02)	14(25.45)	285(21.86)	
7–8 h	784(27.33)	7(12.73)	221(16.95)	
> 8 h	528(18.40)	5(9.09)	120(9.20)	
Body mass index, kg/m²(SD)	24.63(3.15)	19.60(1.76)	24.84(3.01)	< 0.001^#^
Daily steps, *N* (%)				< 0.001^#^
< 4000	300(10.46)	4(7.27)	79(6.06)	
4000–8000	625(21.78)	5(9.09)	174(13.34)	
> 8000	635(22.13)	3(5.45)	121(9.28)	
Alcohol consumption status, *N* (%)				0.175^#^
Drinker	281(9.79)	10(18.18)	132(10.12)	
Non-drinker	2587(90.17)	45(81.82)	1171(89.80)	
Smoking status, *N* (%)				0.140^#^
Smoker	379(13.21)	12(21.82)	157(12.04)	
Non-smoker	2489(86.75)	43(78.18)	1146(87.88)	
Marital status, *N* (%)				0.307^#^
Single	655(22.83)	9(16.36)	316(24.23)	
Non-single	2213(77.13)	46(83.64)	987(75.69)	
Number of chronic diseases, *N* (%)				0.028^#^
0	578(20.15)	19(34.55)	274(21.01)	
1	982(34.23)	15(27.27)	460(35.28)	
2	609(21.23)	6(10.91)	263(20.17)	
> 2	700(24.4)	15(27.27)	307(23.54)	
Number of medications, *N* (%)				0.252^#^
≥ 5	108(3.76)	3(5.45)	41(3.14)	
< 5	2761(96.24)	52(94.55)	1263(96.86)	
Diabetes, *N* (%)	655(22.83)	14(25.45)	309(23.70)	0.502^#^
Hyperlipidemia, *N* (%)	457(15.93)	7(12.73)	207(15.87)	0.797^#^
Coronary heart disease, *N* (%)	366(12.76)	6(10.91)	155(11.89)	0.374^#^
Respiratory disease, *N* (%)	85(2.96)	2(3.64)	36(2.76)	0.823^#^
Musculoskeletal disease, *N* (%)	896(31.23)	12(21.82)	398(30.52)	0.200^#^

^*^p value calculated by Student’s t-test, ^#^*p* value calculated by analysis of χ ²tests

**Fig. 1:**
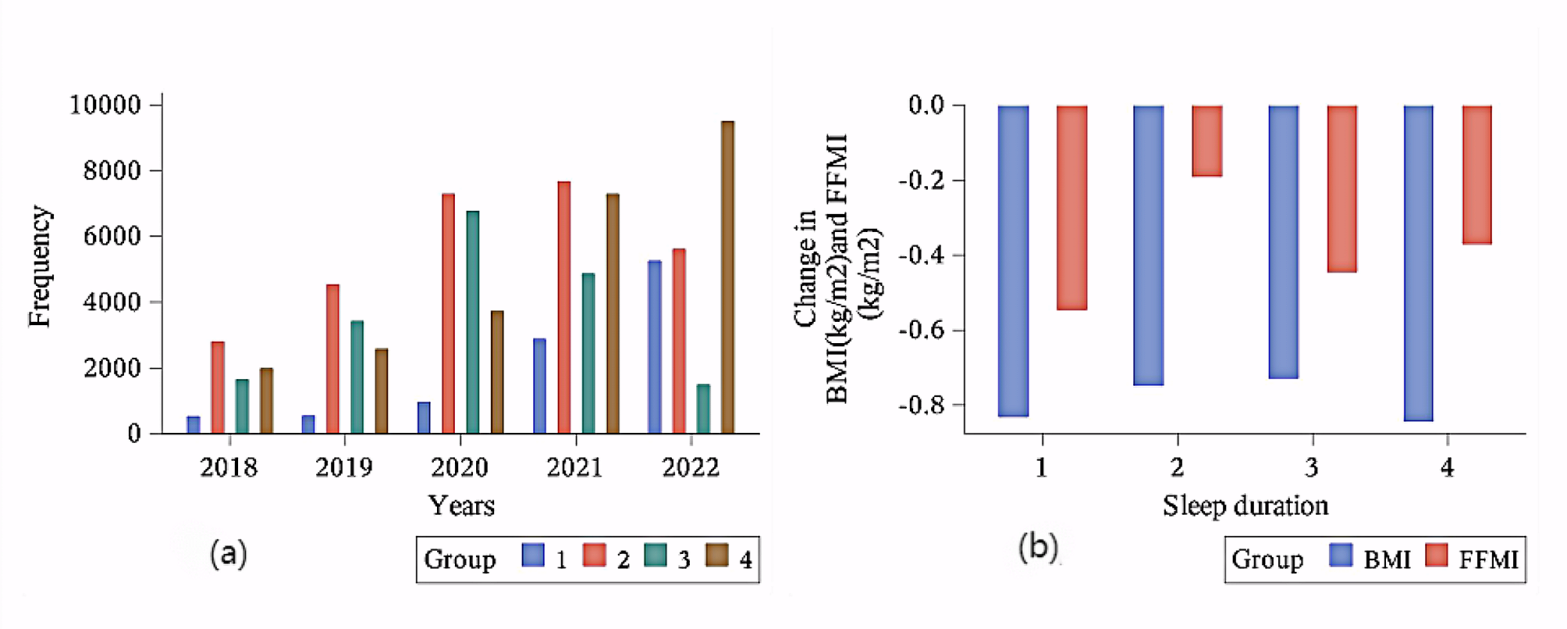
The Frequency of participants’ records of sleep duration (**a**) and mean change in FFMI and BMI for participants in different baseline sleep duration groups (**b**). Sleep duration: 1: <6 h, 2: 7–8 h, 3: ≥8 h, 4:6–7 h

As shown in the figure above (Fig. 1), the frequency of distribution of sleep information recorded in the database for different years, as well as the average change in sleep duration and body mass index for participants with different baseline sleep duration.

**Fig. 2:**
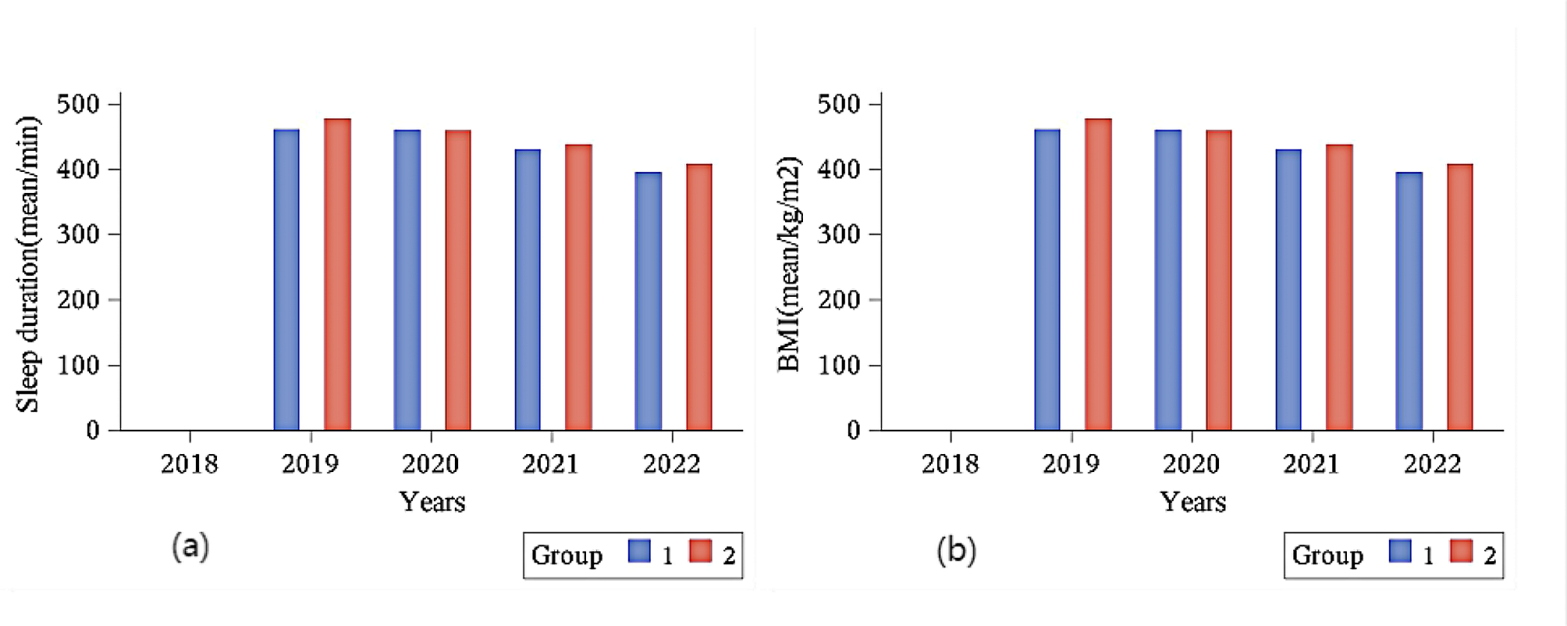
Mean sleep duration(**a**) and mean body mass index(**b**) over time between low and non-low muscle mass. Group: 1: non-low muscle mass; 2: low muscle mass

The figure illustrated the changes in sleep duration(a) and BMI(b) over time in older adults with low and non-low muscle mass (Fig. 2). It showed that older adults with low muscle mass sleep longer than older adults with non-low muscle mass and that both sleep duration and BMI tend to decrease with age.

### Results of multiple imputation

The subset of the mobile support system generated for this study contained 86,037 observations with a missing values rate of about 7% (Table S1). Variables with missing data were daily steps (34.78%), body mass index (30.05%), muscle mass (30.05%), sleep duration (6.92%), age (3.59%), and gender (3.59%), whereas the other variables were almost non-missing. Data were assumed missing at random and non-monotonic missing data pattern (Table S2) and seven datasets were imputed.

A comparison of the distribution of the imputed values with the observed values was showed in Table S3. The means and standard deviations of the continuous variables across the different datasets, even though there was some difference, the overall difference was small, and the distribution was relatively stable. The results of the sensitivity analysis indicated that the results of the multiple repetitions of the estimation process was stable. (Table S4).

### Longitudinal association between sleep duration and low muscle mass

We used sleep duration as the independent variable and muscle mass as the dichotomous response variable, and detailed assignments of the other metrics are shown in Table S5.

The relationship between sleep duration and low muscle mass in different lag structures was showed in Table 2. The results in the different lag structures showed a statistically significant effect of sleeping more than 8 h on low muscle mass. (*p < 0.05*) In the single-month lag model, the effect of long sleep duration on muscle mass reduction became progressively more pronounced with increasing time. In the multi-month lag model, the effect of long sleep duration was significantly greater than in the single-month lag model. While all results were statistically significant, it should be noted that the odds ratios associated with excessive sleep duration were not particularly high.

**Table 2:** Relationship between sleep duration and low muscle mass in different lag structures

Model A^*^				Model B^*^		Model C^*^	
Lag months		OR (95%CI)	*p* value	OR (95%CI)	*p* value	OR (95%CI)	*p* value
0	< 6 h	1.009(0.981–1.037)	0.546	1.000(0.969–1.032)	0.999	1.002(0.970–1.035)	0.902
	7–8 h	1.012(0.993–1.031)	0.219	1.016(0.995–1.038)	0.129	1.017(0.996–1.038)	0.116
	> 8 h	1.046(1.016–1.076)	0.003^**^	1.047(1.016–1.079)	0.003^**^	1.046(1.015–1.078)	0.004^**^
	6–7 h	1	REF	1	REF	1	REF
1	< 6 h	1.006(0.980–1.033)	0.645	0.999(0.970–1.03)	0.972	1.002(0.971–1.033)	0.916
	7–8 h	1.015(0.997–1.033)	0.11	1.019(0.999–1.04)	0.066	1.020(1.000-1.040)	0.054
	> 8 h	1.044(1.014–1.074)	0.003^**^	1.048(1.017–1.08)	0.002^**^	1.047(1.016–1.079)	0.003^**^
	6–7 h	1	REF	1	REF	1	REF
2	< 6 h	1.012(0.984–1.04)	0.404	1.009(0.978–1.041)	0.573	1.011(0.979–1.043)	0.514
	7–8 h	1.014(0.996–1.032)	0.138	1.016(0.996–1.037)	0.111	1.017(0.997–1.037)	0.1
	> 8 h	1.043(1.015–1.073)	0.003^**^	1.05(1.019–1.081)	0.001^**^	1.049(1.018–1.081)	0.002^**^
	6–7 h	1	REF	1	REF	1	REF
0–1	< 6 h	1.014(0.985–1.045)	0.346	1.005(0.971–1.04)	0.791	1.006(0.972–1.041)	0.737
	7–8 h	1.012(0.991–1.034)	0.261	1.018(0.994–1.042)	0.149	1.018(0.994–1.043)	0.139
	> 8 h	1.053(1.020–1.086)	0.001^**^	1.054(1.020–1.089)	0.002^**^	1.053(1.019–1.088)	0.002^**^
	6–7 h	1	REF	1	REF	1	REF
0–2	< 6 h	1.014(0.984–1.046)	0.361	1.003(0.967–1.041)	0.857	1.005(0.969–1.043)	0.774
	7–8 h	1.017(0.995–1.04)	0.135	1.019(0.994–1.044)	0.138	1.02(0.995–1.046)	0.117
	> 8 h	1.049(1.016–1.083)	0.003^**^	1.053(1.018–1.088)	0.002^**^	1.052(1.017–1.088)	0.003^**^
	6–7 h	1	REF	1	REF	1	REF
1–2	< 6 h	1.014(0.984–1.044)	0.372	1.004(0.970–1.039)	0.83	1.005(0.971–1.041)	0.773
	7–8 h	1.016(0.996–1.038)	0.123	1.019(0.996–1.043)	0.107	1.02(0.996–1.044)	0.098
	> 8 h	1.05(1.019–1.082)	0.001^**^	1.054(1.020–1.088)	0.001^**^	1.053(1.019–1.088)	0.002^**^
	6–7 h	1	REF	1	REF	1	REF

*Model A not adjusted for other covariates; *Model B adjusted for age, sex and BMI; *Model C is model B plus adjustment for smoking status, alcohol consumption status, marital status, daily steps, number of chronic diseases, number of medications, Diabetes, Hyperlipidemia, Coronary heart disease, Respiratory disease, Musculoskeletal disease; all models adjusted for observation time-sleep duration interaction and observation time

^*^P < 0.05; ^**^P < 0.01; ^***^P < 0.001

In addition, the result showed significant differences between gender and body mass index and low muscle mass, respectively. (Table S6).

**Fig. 3:**
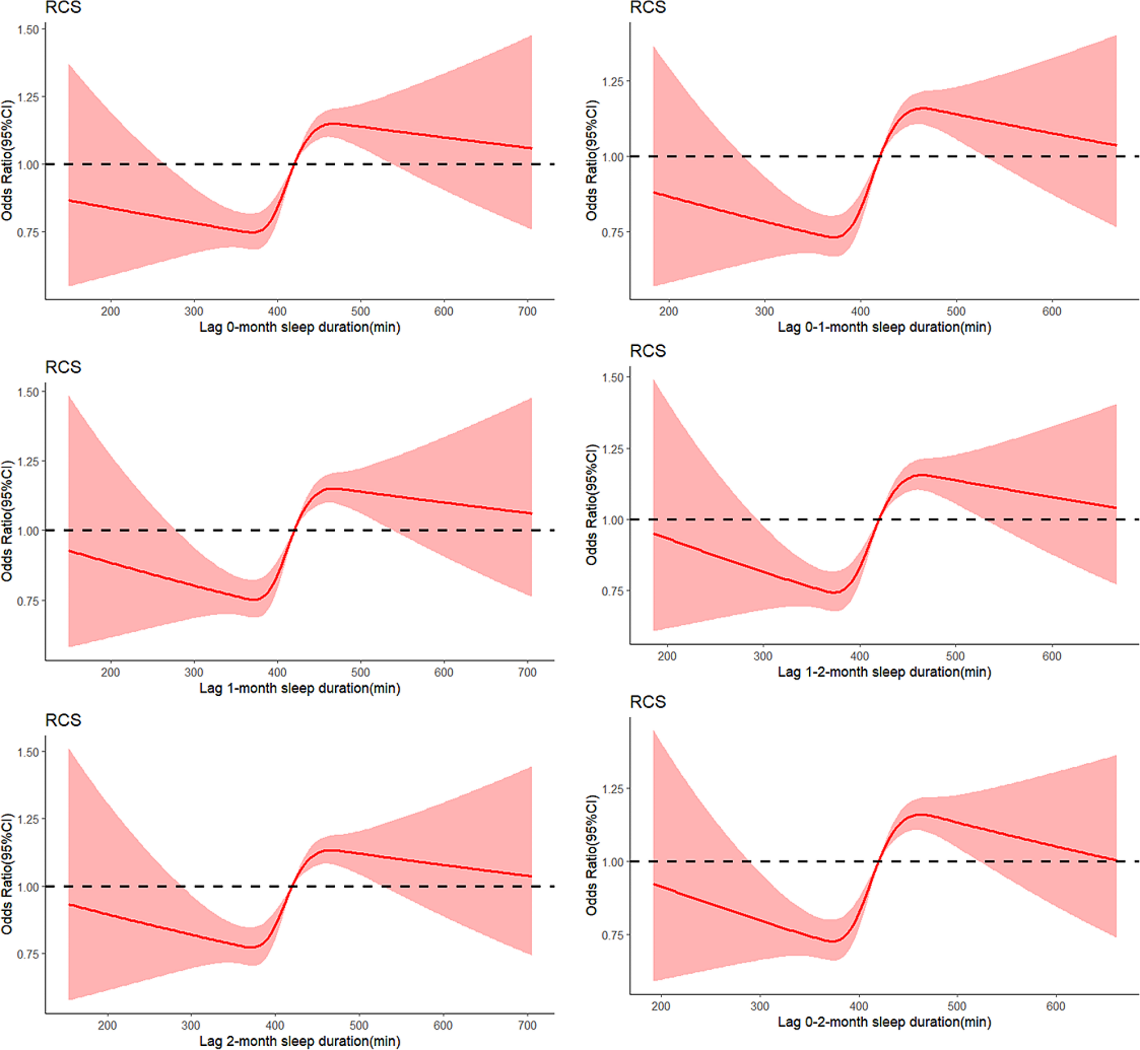
The exposure-response curve of sleep duration and low muscle mass. The x-axis is the lag 0, 1, 2, 0–1, 0–2, 1-2-month sleep duration. Y-axis is the odds ratio, after adjusting for sex, age, smoking status, marital status, number of chronic diseases, number of medications taken, diabetes, coronary heart disease, respiratory disease, and musculoskeletal disease, drinking status, hyperlipidemia, and observation time, is shown by the red solid line, and Light red areas represent 95% confidence intervals

There was a nonlinear correlation between sleep duration and low muscle mass onset (*P* < 0.001). The relationship of the sleep duration and low muscle mass exposure-dose curves for the six lag structures showed that the risk of low muscle mass incidence decreased progressively with increasing sleep duration when the sleep duration was less than 420 minutes, increased with increasing time after reaching a minimum at 370–374 minutes, and decreased slowly with increasing time after reaching a maximum at 463–467 minutes (Fig. 3).

### Subgroup analyses

**Fig. 4:**
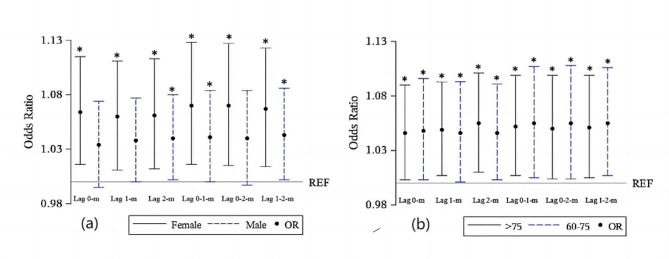
The odds radio and 95% CI for the correlation between more than 8 h of sleep duration (adding a time-sleep interaction term) and muscle mass, stratified by gender (**a**) and age (**b**). ^*^P < 0.05

In subgroup analyses (Fig. 4), significant correlations between long sleep duration and low muscle mass were found only in males at lags of 2 months, 0–1 month, and 1–2 months, whereas in the remaining subgroups significant correlations were found for each sleep lag construct. The effect of long sleep duration on muscle mass loss was significantly greater in females than in males and greater in the multi-month model than in the single-month model. In the age subgroups, the effect of multi-month cumulative long sleep duration on low muscle mass was lower in the over-75 than in the under-75 age group, whereas in the one-month lag structure this effect was lower only in the lag 0-month than in the under-75 age group. As the subgroup analyses showed, while some results were statistically significant, the odds ratios were not high.

## Discussion

Our study was a longitudinal study based on real data from community-dwelling older adults managed on a cloud-based platform. We examined the linear and nonlinear relationships between sleep duration and low muscle mass in older adults over the age of 60. This finding indicated that there was a potential association between sustained sleep duration of more than 8 h and loss of muscle mass, and that this effect became apparent gradually over time. The effect of long sleep duration was also clearly observed in women and older adults of different ages. In all age groups, the effect of a cumulative multi-month lag in sleep duration did not show a greater effect with advanced age, rather it was more pronounced with a single-month lag. However, this potential correlation was not strong, reflecting the fact that the reduction in muscle mass is a multifactorial process, and that longer sleep duration may be a contributing factor, but not a major influence. Therefore, while longer sleep duration may be associated with reduced muscle mass, it is important to interpret these findings in the context of wider health factors and to see it as one of the ways to understand and respond to changes in muscle mass in older people.

Our assessment performed with generalized estimating equations indicated a potential association between sustained sleep of 8 h or more and the progression of low muscle mass in older adults. There are not many studies on the dose-response relationship between sleep duration and low muscle mass in older adults. However, some studies [10, 11, 28]have shown a nonlinear relationship between sleep duration and low muscle mass or sarcopenia in older adults. Hence, we also further verified the relationship between sleep duration and low muscle mass using a restricted cubic spline curve, which showed that when sleep duration was less than 7 h, the risk of developing sarcopenia decreased with longer sleep duration. Nonetheless, when sleep duration exceeded 7 h, sleep duration was associated with the development of hypomuscular mass disorder. This suggested that habitual long sleep duration in older adults is detrimental to muscle mass loss. This result was consistent with other longitudinal studies [19, 20].A longitudinal study [21] of Chinese older adults found that both longer and shorter sleep duration were associated with muscle loss. The difference was that the study was conducted with older adults in rural and suburban China, whereas our study focused on community-based older adults in a large city. Rural and urban older adults have different lifestyles and environments, and these differences make thresholds for low muscle mass inconsistent. In addition, we examined the effect of lagged sleep duration at different periods in the past three months on low muscle mass, whereas most of the other studies focused on short-term sleep duration, such as the past month and seven days, and were mostly based on self-reporting [17, 19] with some recall bias.

Surprisingly, our study found that men were more likely to experience a loss of muscle mass, and older women were instead more likely to experience this after accounting for sleep duration. The results vary from study to study due to different hormone levels, sample sizes, and definitions of muscle mass in different genders [29].However, current research generally recognizes men as a risk factor for muscle mass loss [30–32].When considering sleep duration as a factor, a systematic review concluded that longer sleep duration is more likely to be associated with sarcopenia in older adults, especially women [33]. In contrast, another longitudinal study [20] showed that long sleep duration was associated with low muscle mass only in community-based male older adults. The reason for the inconsistency with previous findings may be that most of the participants in our study were women, and although the onset and rate of muscle mass loss in men is earlier than that in women, changes in hormone levels [5, 34] due to menopause in women accelerate bone loss and muscle decay, and female older adults instead lose more muscle mass in later life [35].

It is important to note that aging plays an important role in muscle mass loss [16], which is mainly due to muscle fiber atrophy and lack of exercise [2, 4, 6]. Aging, which is responsible for chronic diseases, also leads to a faster loss of muscle mass in older adults than in younger adults. Our study found that the cumulative effect of multiple months of sleep on low muscle mass was stronger than that of a single month of sleep, so we must also be wary of older adults who are chronically drowsy for more than a month, especially in the age group of 60–75 years.

Unfortunately sleep structure and duration change with age [12]: either too much or too little persistent nocturnal sleep leads to circadian rhythm disturbances [12, 36], hormone secretion [5, 12, 16, 37] and altered body metabolism [38, 39]. Sleep duration contributes to the loss of muscle mass mainly by affecting the balance of muscle protein synthesis, catabolism and metabolism [6, 12, 37].For example: decreased secretion of growth hormone, testosterone and Insulin-like Growth Factor 1(IGF-1), and insulin resistance inhibit the activity of the Insulin-like Growth Factor 1 (IGF-1)/Phosphoinositide 3-Kinase (PI3K)/Protein Kinase B (Akt) and the mechanistic Target of Rapamycin (mTOR), which reduces muscle protein synthesis and promotes the secretion of Muscle Growth Inhibitory Factor (MGIF) and the expression of Regulated in development and DNA damage responses 1 (REDD1);Increased secretion of cortisol inhibits mTOR activity, promotes REDD1 expression to reduce muscle protein synthesis, and enhances the effects of Atrogin-1 and Muscle RING-finger protein-1 (MuRF-1) through activation of Forkhead box O (FOXO), leading to muscle protein degradation; Chronic inflammation [5, 16, 40], as indicated by elevated levels of pro-inflammatory markers such as c-reactive protein and Interleukin- 6(IL-6), affects muscle anabolism and catabolism. In addition, excessive sleep, and prolonged bed rest [13] lead to reduced daytime physical activity and the effects of chronic diseases.

This study revealed a causal relationship between sleep duration at different lags and low muscle mass in older adults, and the data from these studies are from real-world, long-term follow-up records, allowing for better dynamic analysis of observational variables to demonstrate the reliability of our findings. However, there were some limitations of this study: firstly, we were unable to assess other potential confounders such as information reflecting social status and dietary information; and secondly, medical and medication histories were collected through questionnaires rather than obtained by reviewing medical records.

Sleep duration is an important characteristic of sleep, and measuring the association between sleep and muscle in older adults should consider not only the effect of total sleep duration, but also the effect of other characteristics such as sleep quality and sleep efficiency [41]. Therefore, further future research would be to explore the association between different sleep characteristics and muscle mass after fully considering other covariates.

## Conclusion

Sleep duration in older adults may reflect their physical health to some extent. Our study confirmed a longitudinal association between long sleep duration and low muscle mass in the last three months, especially in older women. We should be alert to older adults who habitually maintain a nightly sleep duration of 8 h or more, which is important to promote their healthy aging.

### Electronic supplementary material

Below is the link to the electronic supplementary material.

Supplementary Material 1

## Data Availability

The data that support the findings of this study are available on request from the corresponding author, [Xiaotong Dai], upon reasonable request.
