# Supplementary material for: Sleeping more than 8 h: a silent factor contributing to decreased muscle mass in Chinese community-dwelling older adults
Source: BMC Public Health. 2024 May 6;24:1246. doi: 10.1186/s12889-024-18520-y (PMC11075226; doi:10.1186/s12889-024-18520-y)
Supplement: Supplementary file 1 — Supplementary Material 1 [file 12889_2024_18520_MOESM1_ESM.docx]

**Supplementary questionnaire:**

**Questionnaire**

**Please read the following entries carefully and fill in the information in " " and the corresponding option "√" according to your actual situation. This survey will cover your health condition, lifestyle and basic personal information in the past month. Your information will be kept strictly confidential and used only for research purposes. Your participation is very important to us and we appreciate your cooperation!**

Please indicate your basic information:

Gender: [ ] Male [ ] Female [ ] Other

Date of Birth:

Age

Nationality:

Q1: How is your current marital status?

1) Single

2) Married

3) Divorced

4) Widowed

Q2: Do you currently smoke?

1)Never

2)Yes

3)Former

Q3: Do you currently drink alcohol?

1)Never

2)Yes

3)Former

Q4: How is your current work status?

1) Work

2) Retired

Q5: What is your level of education?

1) Elementary school

2) Junior high school

3) Senior high school/Technical secondary school

4) University/Junior college and above

Q6: What is your taste preference for meals?

1) Salty diet

2) Regular diet

3) Bland diet

Q7: Have you ever had or do you currently have any of the following diseases?

1) Diabetes

2) Hypertension

3) Hyperlipidemia

4) Coronary heart disease

5) Stroke

6) Respiratory sleep apnea syndrome

7) Chronic kidney disease

8) Parkinsonian syndrome

9) Chronic obstructive pulmonary disease

10) Bronchial asthma

11) Arthritis

12) Osteoporosis

13) Peptic ulcer

14) Cataract Fracture

15) Tumor

16) Heart disease

17) None

Q8: Have you ever taken or are you taking medication for any of the conditions you selected in Q7?

1) Yes

2) No, which diseases are not treated with medication

**Table S1. Missing information of original dataset**

| Variables | Number of observations | Number of missing observations | Missing percent (%) |
| --- | --- | --- | --- |
| Daily steps | 59966 | 31980 | 34.78 |
| Body mass index | 64317 | 27629 | 30.05 |
| Low muscle mass | 64317 | 27629 | 30.05 |
| Sleep duration | 85585 | 6361 | 6.92 |
| Age | 88642 | 3304 | 3.59 |
| Sex | 88642 | 3304 | 3.59 |
| Alcohol consumption status | 91935 | 11 | 0.01 |
| Marital status | 91935 | 11 | 0.01 |
| Smoking status | 91935 | 11 | 0.01 |
| Diabetes | 91946 | 0 | 0 |
| Hyperlipidemia | 91946 | 0 | 0 |
| Coronary heart disease | 91946 | 0 | 0 |
| Respiratory disease | 91946 | 0 | 0 |
| Musculoskeletal disease | 91946 | 0 | 0 |
| Number of chronic diseases | 91946 | 0 | 0 |
| Number of medications | 91946 | 0 | 0 |
| Observation time | 91946 | 0 | 0 |

**Table S2. Missing data pattern information.**

| Missing Data Patterns | | | | | | | | | | | | |
| --- | --- | --- | --- | --- | --- | --- | --- | --- | --- | --- | --- | --- |
| Group | BMI | Daily steps | Smoking Status | Marital Status | Number of Chronic Diseases | Number of Medications | Diabetes | Coronary Heart Disease | Respiratory Diseases | Musculoskeletal Diseases | Freq | Percent |
|  |  |  |  |  |  |  |  |  |  |  |  |  |
| 1 | X | X | X | X | X | X | X | X | X | X | 39450 | 42.91 |
| 2 | X | X | . | . | X | X | X | X | X | X | 2 | 0.00 |
| 3 | X | . | X | X | X | X | X | X | X | X | 24856 | 27.03 |
| 4 | X | . | . | . | X | X | X | X | X | X | 9 | 0.01 |
| 5 | . | X | X | X | X | X | X | X | X | X | 20514 | 22.31 |
| 6 | . | . | X | X | X | X | X | X | X | X | 7115 | 7.74 |

| Missing Data Patterns | | | | | | | | | | |
| --- | --- | --- | --- | --- | --- | --- | --- | --- | --- | --- |
| Group | Group Means | | | | | | | | | |
|  | BMI | Daily steps | Smoking status | Marital Status | Number of chronic diseases | Number of medications | Diabetes | Coronary heart disease | Respiratory disease | Musculoskeletal disease |
| 1 | 23.830497 | 7117.321787 | 1.856730 | 1.769810 | 2.306565 | 1.959163 | 1.780025 | 1.865932 | 1.971381 | 1.676755 |
| 2 | 23.283407 | 5192.537500 | . | . | 4.000000 | 2.000000 | 2.000000 | 2.000000 | 2.000000 | 2.000000 |
| 3 | 23.987503 | . | 1.865465 | 1.772127 | 2.308215 | 1.958239 | 1.776955 | 1.863735 | 1.969706 | 1.665111 |
| 4 | 23.355939 | . | . | . | 4.000000 | 2.000000 | 2.000000 | 2.000000 | 2.000000 | 2.000000 |
| 5 | . | 7117.131715 | 1.859121 | 1.773618 | 2.361899 | 1.960417 | 1.787657 | 1.864385 | 1.971678 | 1.679682 |
| 6 | . | . | 1.879129 | 1.747997 | 2.426564 | 1.960365 | 1.799578 | 1.883204 | 1.974139 | 1.699789 |

**Table S3. Distribution of variables in the pre- and post-fill data sets.**

| Variables | No. | N | Mean | SD | No. | N | Mean | SD |
| --- | --- | --- | --- | --- | --- | --- | --- | --- |
| Daily steps | 0 | 59966 | 7117.19 | 3557.07 | 1 | 91946 | 7105.48 | 3553.47 |
| BMI | 0 | 64317 | 23.89 | 2.87 | 1 | 91946 | 23.88 | 2.87 |
| Age | 0 | 88642 | 73.11 | 6.37 | 1 | 88642 | 73.11 | 6.37 |
| Observation time | 0 | 91946 | 19.10 | 14.08 | 1 | 91946 | 19.10 | 14.08 |
| Sex | 0 | 88642 |  |  | 1 | 88642 |  |  |
| Alcohol consumption status | 0 | 91935 |  |  | 1 | 91935 |  |  |
| Marital status | 0 | 91935 |  |  | 1 | 91946 |  |  |
| Smoking status | 0 | 91935 |  |  | 1 | 91946 |  |  |
| Diabetes | 0 | 91946 |  |  | 1 | 91946 |  |  |
| Hyperlipidemia | 0 | 91946 |  |  | 1 | 91946 |  |  |
| Coronary Heart Disease | 0 | 91946 |  |  | 1 | 91946 |  |  |
| Respiratory Diseases | 0 | 91946 |  |  | 1 | 91946 |  |  |
| Musculoskeletal Diseases | 0 | 91946 |  |  | 1 | 91946 |  |  |
| Number of Chronic Diseases | 0 | 91946 |  |  | 1 | 91946 |  |  |
| Number of Medications | 0 | 91946 |  |  | 1 | 91946 |  |  |

| Variables | No. | N | Mean | SD | No. | N | Mean | SD |
| --- | --- | --- | --- | --- | --- | --- | --- | --- |
| Daily steps | 2 | 91946 | 7108.94 | 3554.37 | 3 | 91946 | 7107.99 | 3561.17 |
| BMI | 2 | 91946 | 23.89 | 2.87 | 3 | 91946 | 23.87 | 2.87 |
| Age | 2 | 88642 | 73.11 | 6.37 | 3 | 88642 | 73.11 | 6.37 |
| Observation time | 2 | 91946 | 19.10 | 14.08 | 3 | 91946 | 19.10 | 14.08 |
| Sex | 2 | 88642 |  |  | 3 | 88642 |  |  |
| Alcohol consumption status | 2 | 91935 |  |  | 3 | 91935 |  |  |
| Marital status | 2 | 91946 |  |  | 3 | 91946 |  |  |
| Smoking status | 2 | 91946 |  |  | 3 | 91946 |  |  |
| Diabetes | 2 | 91946 |  |  | 3 | 91946 |  |  |
| Hyperlipidemia | 2 | 91946 |  |  | 3 | 91946 |  |  |
| Coronary heart disease | 2 | 91946 |  |  | 3 | 91946 |  |  |
| Respiratory disease | 2 | 91946 |  |  | 3 | 91946 |  |  |
| Musculoskeletal disease | 2 | 91946 |  |  | 3 | 91946 |  |  |
| Number of chronic diseases | 2 | 91946 |  |  | 3 | 91946 |  |  |
| Number of medications | 2 | 91946 |  |  | 3 | 91946 |  |  |

| Variables | No. | N | Mean | SD | No. | N | Mean | SD |
| --- | --- | --- | --- | --- | --- | --- | --- | --- |
| Daily steps | 4 | 91946 | 7122.48 | 3551.15 | 5 | 91946 | 7105.75 | 3568.66 |
| BMI | 4 | 91946 | 23.89 | 2.86 | 5 | 91946 | 23.87 | 2.87 |
| Age | 4 | 88642 | 73.11 | 6.37 | 5 | 88642 | 73.11 | 6.37 |
| Observation time | 4 | 91946 | 19.10 | 14.08 | 5 | 91946 | 19.10 | 14.08 |
| Sex | 4 | 88642 |  |  | 5 | 88642 |  |  |
| Alcohol consumption status | 4 | 91935 |  |  | 5 | 91935 |  |  |
| Marital status | 4 | 91946 |  |  | 5 | 91946 |  |  |
| Smoking status | 4 | 91946 |  |  | 5 | 91946 |  |  |
| Diabetes | 4 | 91946 |  |  | 5 | 91946 |  |  |
| Hyperlipidemia | 4 | 91946 |  |  | 5 | 91946 |  |  |
| Coronary heart disease | 4 | 91946 |  |  | 5 | 91946 |  |  |
| Respiratory disease | 4 | 91946 |  |  | 5 | 91946 |  |  |
| Musculoskeletal disease | 4 | 91946 |  |  | 5 | 91946 |  |  |
| Number of chronic diseases | 4 | 91946 |  |  | 5 | 91946 |  |  |
| Number of medications | 4 | 91946 |  |  | 5 | 91946 |  |  |

| Variables | No. | N | Mean | SD | No. | N | Mean | SD |
| --- | --- | --- | --- | --- | --- | --- | --- | --- |
| Daily steps | 6 | 91946 | 7119.15 | 3558.11 | 7 | 91946 | 7119.84 | 3556.16 |
| BMI | 6 | 91946 | 23.89 | 2.87 | 7 | 91946 | 23.90 | 2.87 |
| Age | 6 | 88642 | 73.11 | 6.37 | 7 | 88642 | 73.11 | 6.37 |
| Observation time | 6 | 91946 | 19.10 | 14.08 | 7 | 91946 | 19.10 | 14.08 |
| Sex | 6 | 88642 |  |  | 7 | 88642 |  |  |
| Alcohol consumption status | 6 | 91935 |  |  | 7 | 91935 |  |  |
| Marital status | 6 | 91946 |  |  | 7 | 91946 |  |  |
| Smoking status | 6 | 91946 |  |  | 7 | 91946 |  |  |
| Diabetes | 6 | 91946 |  |  | 7 | 91946 |  |  |
| Hyperlipidemia | 6 | 91946 |  |  | 7 | 91946 |  |  |
| Coronary heart disease | 6 | 91946 |  |  | 7 | 91946 |  |  |
| Respiratory disease | 6 | 91946 |  |  | 7 | 91946 |  |  |
| Musculoskeletal disease | 6 | 91946 |  |  | 7 | 91946 |  |  |
| Number of chronic diseases | 6 | 91946 |  |  | 7 | 91946 |  |  |
| Number of medications | 6 | 91946 |  |  | 7 | 91946 |  |  |

**Table S4. Comparison of OR (95%CI) and *p* value for the dataset before and after filling**

|  |  | Lag 0-month |  | Lag 1-month |  |
| --- | --- | --- | --- | --- | --- |
| Parameter |  | OR（95%CI） | *p* value | OR（95%CI） | *p* value |
| Intercept |  | 0.067(0.007-0.632) | 0.018^*^ | 0.074(0.008-0.692) | 0.022^*^ |
| Sleep duration | <6h | 0.781(0.166-3.682) | 0.754 | 0.827(0.187-3.647) | 0.802 |
|  | 7-8h | 0.597(0.240-1.485) | 0.267 | 0.523(0.218-1.255) | 0.147 |
|  | >8h | 0.214(0.063-0.730) | 0.014^*^ | 0.197(0.058-0.668) | 0.009^**^ |
|  | 6-7h | 1 | REF | 1 | REF |
| time |  | 0.968(0.95-0.985) | <0.001^***^ | 0.965(0.948-0.983) | <0.001^***^ |
| Observation time*sleep duration | <6h | 1.002(0.97-1.035) | 0.902 | 1.002(0.971-1.033) | 0.916 |
|  | 7-8h | 1.017(0.996-1.038) | 0.116 | 1.02(1.000-1.040) | 0.054 |
|  | >8h | 1.046(1.015-1.078) | 0.004^**^ | 1.047(1.016-1.079) | 0.003^**^ |
|  | 6-7h | 1 | REF | 1 | REF |
| Age |  | 1.02(0.994-1.048) | 0.135 | 1.02(0.994-1.048) | 0.137 |
| Sex | Female | 0.286(0.188-0.434) | <0.001^***^ | 0.293(0.192-0.446) | <0.001^***^ |
|  | Male | 1 | REF | 1 | REF |
| BMI | <18.5 | 100.077(66.774-149.99) | <0.001^***^ | 101.912(67.695-153.425) | <0.001^***^ |
|  | ≥18.5 | 1 | REF | 1 | REF |
| Daily steps | <4000 | 1.088(0.808-1.465) | 0.580 | 1.111(0.827-1.494) | 0.484 |
|  | 4000-8000 | 1.131(0.906-1.411) | 0.277 | 1.149(0.92-1.434) | 0.221 |
|  | >8000 | 1 | REF | 1 | REF |
| Alcohol Consumption status | Drinker | 1.255(0.768-2.05) | 0.365 | 1.255(0.763-2.064) | 0.371 |
|  | Non-drinker | 1 | REF | 1 | REF |
| Smoking status | Smoker | 0.868(0.547-1.379) | 0.549 | 0.887(0.554-1.421) | 0.619 |
|  | Non-smoker | 1 | REF | 1 | REF |
| Marital status | Single | 0.737(0.468-1.163) | 0.190 | 0.73(0.461-1.155) | 0.179 |
|  | Non-single | 1 | REF | 1 | REF |
| Number of chronic diseases | 1 | 0.769(0.476-1.244) | 0.285 | 0.765(0.473-1.236) | 0.273 |
|  | 2 | 0.703(0.384-1.287) | 0.254 | 0.702(0.384-1.286) | 0.252 |
|  | ≥2 | 0.814(0.373-1.777) | 0.606 | 0.8(0.362-1.766) | 0.580 |
|  | 0 | 1 | REF | 1 | REF |
| Number of medications | ≥5 | 1.721(0.632-4.686) | 0.288 | 1.784(0.649-4.905) | 0.262 |
|  | <5 | 1 | REF | 1 | REF |
| Diabetes | Yes | 0.773(0.477-1.252) | 0.295 | 0.766(0.470-1.248) | 0.285 |
|  | No | 1 | REF | 1 | REF |
| Hyperlipidemia | Yes | 0.73(0.390-1.366) | 0.324 | 0.736(0.391-1.386) | 0.342 |
|  | No | 1 | REF | 1 | REF |
| Coronary heart disease | Yes | 0.569(0.288-1.122) | 0.104 | 0.544(0.272-1.087) | 0.085 |
|  | No | 1 | REF | 1 | REF |
| Respiratory disease | Yes | 1.025(0.557-1.889) | 0.936 | 1.039(0.558-1.937) | 0.904 |
|  | No | 1 | REF | 1 | REF |
| Musculoskeletal disease | Yes | 1.017(0.646-1.602) | 0.941 | 1.008(0.637-1.595) | 0.973 |
|  | No | 1 | REF | 1 | REF |

^*^*P* <0.05; ^**^*P* <0.01; ^***^*P* <0.001

|  |  | Lag 2-months |  | Lag 0-1-months |  |
| --- | --- | --- | --- | --- | --- |
| Parameter |  | OR（95%CI） | *p* value | OR（95%CI） | *p* value |
| Intercept |  | 0.071(0.007-0.691) | 0.023^*^ | 0.081(0.008-0.820) | 0.033^*^ |
| Sleep duration | <6h | 0.523(0.109-2.496) | 0.416 | 0.628(0.121-3.266) | 0.581 |
|  | 7-8h | 0.551(0.228-1.332) | 0.186 | 0.54(0.190-1.537) | 0.248 |
|  | >8h | 0.172(0.05-0.592) | 0.005^**^ | 0.162(0.043-0.616) | 0.008^**^ |
|  | 6-7h | 1 | REF | 1 | REF |
| time |  | 0.965(0.948-0.983) | <0.001^***^ | 0.965(0.946-0.985) | <0.001^***^ |
| Observation time*sleep duration | <6h | 1.011(0.979-1.043) | 0.514 | 1.006(0.972-1.041) | 0.737 |
|  | 7-8h | 1.017(0.997-1.037) | 0.100 | 1.018(0.994-1.043) | 0.139 |
|  | >8h | 1.049(1.018-1.081) | 0.002^**^ | 1.053(1.019-1.088) | 0.002^**^ |
|  | 6-7h | 1 | REF | 1 | REF |
| Age |  | 1.022(0.994-1.050) | 0.122 | 1.02(0.992-1.048) | 0.158 |
| Sex | Female | 0.299(0.195-0.458) | <0.001^***^ | 0.287(0.187-0.44) | <0.001^***^ |
|  | Male | 1 | REF | 1 | REF |
| BMI | <18.5 | 100.251(66.693-150.695) | <0.001^***^ | 101.525(67.175-153.44) | <0.001^***^ |
|  | ≥18.5 | 1 | REF | 1 | REF |
| Daily steps | <4000 | 1.1(0.815-1.485) | 0.531 | 1.065(0.783-1.449) | 0.689 |
|  | 4000-8000 | 1.143(0.914-1.429) | 0.242 | 1.146(0.912-1.439) | 0.243 |
|  | >8000 | 1 | REF | 1 | REF |
| Alcohol Consumption status | Drinker | 1.288(0.783-2.121) | 0.319 | 1.245(0.755-2.055) | 0.390 |
|  | Non-drinker | 1 | REF | 1 | REF |
| Smoking status | Smoker | 0.891(0.554-1.434) | 0.636 | 0.875(0.546-1.402) | 0.578 |
|  | Non-smoker | 1 | REF | 1 | REF |
| Marital status | Single | 0.723(0.456-1.145) | 0.166 | 0.744(0.47-1.178) | 0.207 |
|  | Non-single | 1 | REF | 1 | REF |
| Number of chronic diseases | 1 | 0.751(0.463-1.218) | 0.246 | 0.755(0.462-1.232) | 0.261 |
|  | 2 | 0.688(0.372-1.272) | 0.233 | 0.683(0.371-1.255) | 0.220 |
|  | ≥2 | 0.783(0.352-1.742) | 0.549 | 0.793(0.360-1.750) | 0.566 |
|  | 0 | 1 | REF | 1 | REF |
| Number of medications | ≥5 | 1.824(0.649-5.123) | 0.254 | 1.809(0.653-5.014) | 0.254 |
|  | <5 | 1 | REF | 1 | REF |
| Diabetes | Yes | 0.75(0.456-1.234) | 0.257 | 0.736(0.445-1.216) | 0.232 |
|  | No | 1 | REF | 1 | REF |
| Hyperlipidemia | Yes | 0.721(0.378-1.375) | 0.320 | 0.728(0.384-1.379) | 0.330 |
|  | No | 1 | REF | 1 | REF |
| Coronary heart disease | Yes | 0.536(0.266-1.08) | 0.081 | 0.545(0.272-1.090) | 0.086 |
|  | No | 1 | REF | 1 | REF |
| Respiratory disease | Yes | 1.047(0.562-1.950) | 0.885 | 1.017(0.537-1.926) | 0.958 |
|  | No | 1 | REF | 1 | REF |
| Musculoskeletal disease | Yes | 0.99(0.62-1.582) | 0.968 | 1.031(0.657-1.620) | 0.893 |
|  | No | 1 | REF | 1 | REF |

^*^*P* <0.05; ^**^*P* <0.01; ^***^*P* <0.001

|  |  | Lag 0-2-months |  | Lag 1-2-months |  |
| --- | --- | --- | --- | --- | --- |
| Parameter |  | OR（95%CI） | *p* value | OR（95%CI） | *p* value |
| Intercept |  | 0.08(0.008-0.847) | 0.036 | 0.078(0.008-0.804) | 0.032 |
| Sleep duration | <6h | 0.648(0.108-3.892) | 0.636 | 0.688(0.13-3.653) | 0.661 |
|  | 7-8h | 0.487(0.161-1.471) | 0.202 | 0.506(0.181-1.413) | 0.193 |
|  | >8h | 0.171(0.043-0.674) | 0.012^*^ | 0.158(0.042-0.599) | 0.007^**^ |
|  | 6-7h | 1 | REF | 1 | REF |
| time |  | 0.966(0.946-0.986) | <0.001^***^ | 0.964(0.945-0.984) | <0.001^***^ |
| Observation time*sleep duration | <6h | 1.005(0.969-1.043) | 0.774 | 1.005(0.971-1.041) | 0.773 |
|  | 7-8h | 1.02(0.995-1.046) | 0.117 | 1.02(0.996-1.044) | 0.098 |
|  | >8h | 1.052(1.017-1.088) | 0.003^**^ | 1.053(1.019-1.088) | 0.002^**^ |
|  | 6-7h | 1 | REF | 1 | REF |
| Age |  | 1.02(0.992-1.049) | 0.171 | 1.02(0.993-1.049) | 0.149 |
| Sex | Female | 0.292(0.188-0.452) | <0.001^***^ | 0.296(0.192-0.455) | <0.001^***^ |
|  | Male | 1 | REF | 1 | REF |
| BMI | <18.5 | 101.005(66.673-153.016) | <0.001^***^ | 101.907(67.328-154.246) | <0.001^***^ |
|  | ≥18.5 | 1 | REF | 1 | REF |
| Daily steps | <4000 | 1.035(0.755-1.418) | 0.831 | 1.067(0.785-1.451) | 0.679 |
|  | 4000-8000 | 1.14(0.907-1.434) | 0.261 | 1.14(0.910-1.428) | 0.254 |
|  | >8000 | 1 | REF | 1 | REF |
| Alcohol Consumption status | Drinker | 1.26(0.759-2.091) | 0.372 | 1.26(0.760-2.087) | 0.370 |
|  | Non-drinker | 1 | REF | 1 | REF |
| Smoking status | Smoker | 0.883(0.547-1.423) | 0.608 | 0.888(0.551-1.431) | 0.625 |
|  | Non-smoker | 1 | REF | 1 | REF |
| Marital status | Single | 0.741(0.465-1.181) | 0.208 | 0.726(0.458-1.15) | 0.173 |
|  | Non-single | 1 | REF | 1 | REF |
| Number of chronic diseases | 1 | 0.742(0.45-1.224) | 0.243 | 0.753(0.460-1.233) | 0.260 |
|  | 2 | 0.654(0.353-1.214) | 0.178 | 0.675(0.365-1.251) | 0.212 |
|  | ≥2 | 0.766(0.344-1.708) | 0.515 | 0.785(0.353-1.746) | 0.553 |
|  | 0 | 1 | REF | 1 | REF |
| Number of medications | ≥5 | 1.862(0.658-5.268) | 0.241 | 1.862(0.662-5.233) | 0.239 |
|  | <5 | 1 | REF | 1 | REF |
| Diabetes | Yes | 0.712(0.423-1.198) | 0.201 | 0.725(0.435-1.207) | 0.216 |
|  | No | 1 | REF | 1 | REF |
| Hyperlipidemia | Yes | 0.733(0.381-1.411) | 0.353 | 0.727(0.381-1.388) | 0.334 |
|  | No | 1 | REF | 1 | REF |
| Coronary heart disease | Yes | 0.537(0.265-1.088) | 0.084 | 0.532(0.262-1.079) | 0.080 |
|  | No | 1 | REF | 1 | REF |
| Respiratory disease | Yes | 1.026(0.536-1.964) | 0.939 | 1.023(0.536-1.954) | 0.945 |
|  | No | 1 | REF | 1 | REF |
| Musculoskeletal disease | Yes | 1.041(0.661-1.639) | 0.862 | 1.016(0.642-1.607) | 0.947 |
|  | No | 1 | REF | 1 | REF |

^*^*P* <0.05; ^**^*P* <0.01; ^***^*P* <0.001

**Table S5. Variable assignment table**

| Factors | Variable | Definition |
| --- | --- | --- |
| Sleep duration | X1 | 1=X1<6h 2=7<X1≤8h 3=X1≥8h 4=6h≤X1≤7h |
| Observation time | X2 | Continuous variable |
| Age | X3 | Continuous variable |
| Sex | X4 | 1=male 0=female |
| Body mass index | X5 | 1=X5<18.5 2=X5≥18.5 |
| Daily steps | X6 | 1= X6＜4000 2=4000<=X6<= 8000 3=X6>8000 |
| Alcohol consumption status | X7 | 1=drink 2=non-drink |
| Smoking status | X8 | 1=smoke 3=non-smoke |
| Marital status | X9 | 1=with a partner 2=no partner |
| Number of chronic diseases | X10 | 1=X10=1 2=X10=2 3=X10>2 4=X10=0 |
| Number of medications | X11 | 1=Yes 2=No |
| Diabetes | X12 | 1=Yes 2=No |
| Hyperlipidemia | X13 | 1=Yes 2=No |
| Coronary heart disease | X14 | 1=Yes 2=No |
| Respiratory disease | X15 | 1=Yes 2=No |
| Musculoskeletal disease | X16 | 1=Yes 2=No |
| Heart disease | X17 | 1=Yes 2=No |
| Low muscle mass | X18 | 1=Yes 2=No |

**Table S6. Relationship between lagged sleep duration and low muscle mass across time.**

|  |  | **Lag 0-month** |  | **Lag 1-month** |  |
| --- | --- | --- | --- | --- | --- |
| Parameter |  | OR（95%CI） | *p* value | OR（95%CI） | *p* -value |
| Intercept |  | 0.067(0.007-0.632) | 0.018^*^ | 0.074(0.008-0.692) | 0.022^*^ |
| Sleep duration | <6h | 0.781(0.166-3.682) | 0.754 | 0.827(0.187-3.647) | 0.802 |
|  | 7-8h | 0.597(0.24-1.485) | 0.267 | 0.523(0.218-1.255) | 0.147 |
|  | >8h | 0.214(0.063-0.73) | 0.014^*^ | 0.197(0.058-0.668) | 0.009^**^ |
|  | 6-7h | 1 | REF | 1 | REF |
| time |  | 0.968(0.950-0.985) | <0.001^***^ | 0.965(0.948-0.983) | <0.001^***^ |
| Observation time*sleep duration | <6h | 1.002(0.970-1.035) | 0.902 | 1.002(0.971-1.033) | 0.916 |
|  | 7-8h | 1.017(0.996-1.038) | 0.116 | 1.02(1-1.04) | 0.054 |
|  | >8h | 1.046(1.015-1.078) | 0.004^**^ | 1.047(1.016-1.079) | 0.003^**^ |
|  | 6-7h | 1 | REF | 1 | REF |
| Age |  | 1.02(0.994-1.048) | 0.135 | 1.02(0.994-1.048) | 0.137 |
| Sex | Female | 0.286(0.188-0.434) | <0.001^***^ | 0.293(0.192-0.446) | <0.001^***^ |
|  | Male | 1 | REF | 1 | REF |
| BMI | <18.5 | 100.077(66.774-149.99) | <0.001^***^ | 101.912(67.695-153.425) | <0.001^***^ |
|  | ≥18.5 | 1 | REF | 1 | REF |
| Daily steps | <4000 | 1.088(0.808-1.465) | 0.580 | 1.111(0.827-1.494) | 0.484 |
|  | 4000-8000 | 1.131(0.906-1.411) | 0.277 | 1.149(0.92-1.434) | 0.221 |
|  | >8000 | 1 | REF | 1 | REF |
| Alcohol Consumption status | Drinker | 1.255(0.768-2.05) | 0.365 | 1.255(0.763-2.064) | 0.371 |
|  | Non-drinker | 1 | REF | 1 | REF |
| Smoking status | Smoker | 0.868(0.547-1.379) | 0.549 | 0.887(0.554-1.421) | 0.619 |
|  | Non-smoker | 1 | REF | 1 | REF |
| Marital status | Single | 0.737(0.468-1.163) | 0.190 | 0.73(0.461-1.155) | 0.179 |
|  | Non-single | 1 | REF | 1 | REF |
| Number of chronic diseases | 1 | 0.769(0.476-1.244) | 0.285 | 0.765(0.473-1.236) | 0.273 |
|  | 2 | 0.703(0.384-1.287) | 0.254 | 0.702(0.384-1.286) | 0.252 |
|  | ≥2 | 0.814(0.373-1.777) | 0.606 | 0.8(0.362-1.766) | 0.580 |
|  | 0 | 1 | REF | 1 | REF |
| Number of medications | ≥5 | 1.721(0.632-4.686) | 0.288 | 1.784(0.649-4.905) | 0.262 |
|  | <5 | REF | REF | REF | REF |
| Diabetes | Yes | 0.773(0.477-1.252) | 0.295 | 0.766(0.470-1.248) | 0.285 |
|  | No | REF | REF | REF | REF |
| Hyperlipidemia | Yes | 0.73(0.390-1.366) | 0.324 | 0.736(0.391-1.386) | 0.342 |
|  | No | REF | REF | REF | REF |
| Coronary heart disease | Yes | 0.569(0.288-1.122) | 0.104 | 0.544(0.272-1.087) | 0.085 |
|  | No | REF | REF | REF | REF |
| Respiratory disease | Yes | 1.025(0.557-1.889) | 0.936 | 1.039(0.558-1.937) | 0.904 |
|  | No | REF | REF | REF | REF |
| Musculoskeletal disease | Yes | 1.017(0.646-1.602) | 0.941 | 1.008(0.637-1.595) | 0.973 |
|  | No | REF | REF | REF | REF |

^*^*P* <0.05; ^**^*P* <0.01; ^***^*P* <0.001

|  |  | **Lag 2-month** |  | Lag 0-1-month |  |
| --- | --- | --- | --- | --- | --- |
| Parameter |  | OR（95%CI） | ***P***-value | OR（95%CI） | ***P***-value |
| Intercept |  | 0.071(0.007-0.691) | 0.023^*^ | 0.081(0.008-0.82) | 0.033^*^ |
| Sleep duration | <6h | 0.523(0.109-2.496) | 0.416 | 0.628(0.121-3.266) | 0.581 |
|  | 7-8h | 0.551(0.228-1.332) | 0.186 | 0.54(0.190-1.537) | 0.248 |
|  | >8h | 0.172(0.050-0.592) | 0.005^**^ | 0.162(0.043-0.616) | 0.008^**^ |
|  | 6-7h | 1 | REF | 1 | REF |
| time |  | 0.965(0.948-0.983) | <0.001^***^ | 0.965(0.946-0.985) | <0.001^***^ |
| Observation time*sleep duration | <6h | 1.011(0.979-1.043) | 0.514 | 1.006(0.972-1.041) | 0.737 |
|  | 7-8h | 1.017(0.997-1.037) | 0.100 | 1.018(0.994-1.043) | 0.139 |
|  | >8h | 1.049(1.018-1.081) | 0.002^**^ | 1.053(1.019-1.088) | 0.002^**^ |
|  | 6-7h | 1 | REF | 1 | REF |
| Age |  | 1.022(0.994-1.05) | 0.122 | 1.02(0.992-1.048) | 0.158 |
| Sex | Female | 0.299(0.195-0.458) | <0.001^***^ | 0.287(0.187-0.440) | <0.001^***^ |
|  | Male | 1 | REF | 1 | REF |
| BMI | <18.5 | 100.251(66.693-150.695) | <0.001^***^ | 101.525(67.175-153.44) | <0.001^***^ |
|  | ≥18.5 | 1 | REF | 1 | REF |
| Daily steps | <4000 | 1.1(0.815-1.485) | 0.531 | 1.065(0.783-1.449) | 0.689 |
|  | 4000-8000 | 1.143(0.914-1.429) | 0.242 | 1.146(0.912-1.439) | 0.243 |
|  | >8000 | 1 | REF | 1 | REF |
| Alcohol Consumption status | Drinker | 1.288(0.783-2.121) | 0.319 | 1.245(0.755-2.055) | 0.390 |
|  | Non-drinker | 1 | REF | 1 | REF |
| Smoking status | Smoker | 0.891(0.554-1.434) | 0.636 | 0.875(0.546-1.402) | 0.578 |
|  | Non-smoker | 1 | REF | 1 | REF |
| Marital status | Single | 0.723(0.456-1.145) | 0.166 | 0.744(0.470-1.178) | 0.207 |
|  | Non-single | 1 | REF | 1 | REF |
| Number of chronic diseases | 1 | 0.751(0.463-1.218) | 0.246 | 0.755(0.462-1.232) | 0.261 |
|  | 2 | 0.688(0.372-1.272) | 0.233 | 0.683(0.371-1.255) | 0.220 |
|  | ≥2 | 0.783(0.352-1.742) | 0.549 | 0.793(0.360-1.75) | 0.566 |
|  | 0 | 1 | REF | 1 | REF |
| Number of medications | ≥5 | 1.824(0.649-5.123) | 0.254 | 1.809(0.653-5.014) | 0.254 |
|  | <5 | 1 | REF | 1 | REF |
| Diabetes | Yes | 0.750(0.456-1.234) | 0.257 | 0.736(0.445-1.216) | 0.232 |
|  | No | 1 | REF | 1 | REF |
| Hyperlipidemia | Yes | 0.721(0.378-1.375) | 0.320 | 0.728(0.384-1.379) | 0.330 |
|  | No | 1 | REF | 1 | REF |
| Coronary heart disease | Yes | 0.536(0.266-1.08) | 0.081 | 0.545(0.272-1.09) | 0.086 |
|  | No | 1 | REF | 1 | REF |
| Respiratory disease | Yes | 1.047(0.562-1.95) | 0.885 | 1.017(0.537-1.926) | 0.958 |
|  | No | 1 | REF | 1 | REF |
| Musculoskeletal disease | Yes | 0.990(0.62-1.582) | 0.968 | 1.031(0.657-1.62) | 0.893 |
|  | No | 1 | REF | 1 | REF |

^*^*P* <0.05; ^**^*P* <0.01; ^***^*P* <0.001

|  |  | Lag 0-1-month |  | Lag 0-2-month |  |
| --- | --- | --- | --- | --- | --- |
| Parameter |  | OR（95%CI） | *p* value | OR（95%CI） | *p* value |
| Intercept |  | 0.081(0.008-0.82) | 0.033^*^ | 0.080(0.008-0.847) | 0.036^*^ |
| Sleep duration | <6h | 0.628(0.121-3.266) | 0.581 | 0.648(0.108-3.892) | 0.636 |
|  | 7-8h | 0.54(0.190-1.537) | 0.248 | 0.487(0.161-1.471) | 0.202 |
|  | >8h | 0.162(0.043-0.616) | 0.008^**^ | 0.171(0.043-0.674) | 0.012^**^ |
|  | 6-7h | 1 | REF | 1 | REF |
| time |  | 0.965(0.946-0.985) | <0.001^***^ | 0.966(0.946-0.986) | <0.001^***^ |
| Observation time*sleep duration | <6h | 1.006(0.972-1.041) | 0.737 | 1.005(0.969-1.043) | 0.774 |
|  | 7-8h | 1.018(0.994-1.043) | 0.139 | 1.020(0.995-1.046) | 0.117 |
|  | >8h | 1.053(1.019-1.088) | 0.002^**^ | 1.052(1.017-1.088) | 0.003^**^ |
|  | 6-7h | 1 | REF | 1 | REF |
| Age |  | 1.02(0.992-1.048) | 0.158 | 1.02(0.992-1.049) | 0.171 |
| Sex | Female | 0.287(0.187-0.440) | <0.001^***^ | 0.292(0.188-0.452) | <0.001^***^ |
|  | Male | 1 | REF | 1 | REF |
| BMI | <18.5 | 101.525(67.175-153.44) | <0.001^***^ | 101.005(66.673-153.016) | <0.001^***^ |
|  | ≥18.5 | 1 | REF | 1 | REF |
| Daily steps | <4000 | 1.065(0.783-1.449) | 0.689 | 1.035(0.755-1.418) | 0.831 |
|  | 4000-8000 | 1.146(0.912-1.439) | 0.243 | 1.140(0.907-1.434) | 0.261 |
|  | >8000 | 1 | REF | 1 | REF |
| Alcohol Consumption status | Drinker | 1.245(0.755-2.055) | 0.39 | 1.260(0.759-2.091) | 0.372 |
|  | Non-drinker | 1 | REF | 1 | REF |
| Smoking status | Smoker | 0.875(0.546-1.402) | 0.578 | 0.883(0.547-1.423) | 0.608 |
|  | Non-smoker | 1 | REF | 1 | REF |
| Marital status | Single | 0.744(0.47-1.178) | 0.207 | 0.741(0.465-1.181) | 0.208 |
|  | Non-single | 1 | REF | 1 | REF |
| Number of chronic diseases | 1 | 0.755(0.462-1.232) | 0.261 | 0.742(0.450-1.224) | 0.243 |
|  | 2 | 0.683(0.371-1.255) | 0.22 | 0.654(0.353-1.214) | 0.178 |
|  | ≥2 | 0.793(0.360-1.75) | 0.566 | 0.766(0.344-1.708) | 0.515 |
|  | 0 | 1 | REF | 1 | REF |
| Number of medications | ≥5 | 1.809(0.653-5.014) | 0.254 | 1.862(0.658-5.268) | 0.241 |
|  | <5 | 1 | REF | 1 | REF |
| Diabetes | Yes | 0.736(0.445-1.216) | 0.232 | 0.712(0.423-1.198) | 0.201 |
|  | No | 1 | REF | 1 | REF |
| Hyperlipidemia | Yes | 0.728(0.384-1.379) | 0.33 | 0.733(0.381-1.411) | 0.353 |
|  | No | 1 | REF | 1 | REF |
| Coronary heart disease | Yes | 0.545(0.272-1.09) | 0.086 | 0.537(0.265-1.088) | 0.084 |
|  | No | 1 | REF | 1 | REF |
| Respiratory disease | Yes | 1.017(0.537-1.926) | 0.958 | 1.026(0.536-1.964) | 0.939 |
|  | No | 1 | REF | 1 | REF |
| Musculoskeletal disease | Yes | 1.031(0.657-1.62) | 0.893 | 1.041(0.661-1.639) | 0.862 |

^*^*P* <0.05; ^**^*P* <0.01; ^***^*P* <0.001
